# Investigating the Genetic Diversity of Hepatitis Delta Virus in Hepatocellular Carcinoma (HCC): Impact on Viral Evolution and Oncogenesis in HCC

**DOI:** 10.3390/v16060817

**Published:** 2024-05-21

**Authors:** Horng-Heng Juang, Chao-Wei Hsu, Kang-Shuo Chang, Shan-Bei Iang, Yang-Hsiang Lin, Mei Chao

**Affiliations:** 1 Department of Anatomy, Graduate Institute of Biomedical Sciences, School of Medicine, Chang Gung University, Taoyuan 333, Taiwan; hhj143@cgu.edu.tw (H.-H.J.); d000016684@cgu.edu.tw (K.-S.C.); 2 Department of Urology, Chang Gung Memorial Hospital at Linkou, Taoyuan 333, Taiwan; 3 Liver Research Center, Department of Gastroenterology and Hepatology, Chang Gung Memorial Hospital at Linkou, Taoyuan 333, Taiwan; hsu2406@cloud.cgmh.org.tw (C.-W.H.); yhlin0621@cgmh.org.tw (Y.-H.L.); 4 School of Medicine, Chang Gung University, Taoyuan 333, Taiwan; 5 Department of Microbiology and Immunology and Division of Microbiology, Graduate Institute of Biomedical Sciences, School of Medicine, Chang Gung University, Taoyuan 333, Taiwan; isbisbisb@yahoo.com.tw

**Keywords:** hepatitis delta virus, hepatocellular carcinoma, RNA recombination, delta antigen, cell migration and invasion

## Abstract

Hepatitis delta virus (HDV), an RNA virus with two forms of the delta antigen (HDAg), relies on hepatitis B virus (HBV) for envelope proteins essential for hepatocyte entry. Hepatocellular carcinoma (HCC) ranks third in global cancer deaths, yet HDV’s involvement remains uncertain. Among 300 HBV-associated HCC serum samples from Taiwan’s National Health Research Institutes, 2.7% (8/300) tested anti-HDV positive, with 62.7% (5/8) of these also HDV RNA positive. Genotyping revealed HDV-2 in one sample, HDV-4 in two, and two samples showed mixed HDV-2/HDV-4 infection with RNA recombination. A mixed-genotype infection revealed novel mutations at the polyadenylation signal, coinciding with the ochre termination codon for the L-HDAg. To delve deeper into the possible oncogenic properties of HDV-2, the predominant genotype in Taiwan, which was previously thought to be less associated with severe disease outcomes, an HDV-2 cDNA clone was isolated from HCC for study. It demonstrated a replication level reaching up to 74% of that observed for a widely used HDV-1 strain in transfected cultured cells. Surprisingly, both forms of HDV-2 HDAg promoted cell migration and invasion, affecting the rearrangement of actin cytoskeleton and the expression of epithelial–mesenchymal transition markers. In summary, this study underscores the prevalence of HDV-2, HDV-4, and their mixed infections in HCC, highlighting the genetic diversity in HCC as well as the potential role of both forms of the HDAg in HCC oncogenesis.

## 1. Introduction

Hepatitis delta virus (HDV) is a satellite virus of hepatitis B virus (HBV), encapsulated by HBV surface antigens (HBsAg), crucial for virion assembly and transmission [1,2]. HDV infection can manifest as co-infection or superinfection. Co-infection involves simultaneous HBV and HDV infection, while superinfection occurs when HDV infects someone already chronically infected with HBV. The precise prevalence of anti-HDV antibodies is still unclear, with various approximations ranging between 5% and 15% among those who test positive for HBsAg [3,4,5]. However, a recent study examining HDV prevalence across 25 countries/territories has revealed a significantly lower anti-HDV prevalence of 2.0% compared to previous estimates [6]. This variability may stem from inconsistent diagnostic criteria across different countries and insufficient screening efforts in many regions. HBV/HDV dual infections represent the most severe form of viral hepatitis, potentially leading to life-threatening fulminant hepatitis, and progression to chronic inflammation, fibrosis, cirrhosis, and hepatocellular carcinoma (HCC) [7,8]. While the World Health Organization has classified HBV and hepatitis C virus (HCV) as human carcinogens, the contribution of HDV to the development of HCC is still a subject of debate. However, chronic HBV/HDV co-infection is known to pose an increased risk of HCC compared to those with chronic HBV mono-infection, indicating a potential association [8,9].

HDV belongs to the negative-sense RNA virus family *Kolmioviridae*. Within the HDV virion, there exists a small (1.7-kb) RNA genome complexed with the sole viral-encoded protein, the delta antigen (HDAg). Both its RNA genome and antigenome exhibit a circular conformation, capable of folding into an unbranched rod-like structure. HDV replication involves the redirection of host DNA-dependent RNA polymerase(s) to transcribe HDV RNA in the nucleus [10,11]. In the replication process, the host-encoded small form of ADAR1 performs amber/W RNA editing on certain antigenomes, leading to the incorporation of an extra 19–20 amino acids at the C terminus of the HDAg [12]. Consequently, the delta antigen is present in two forms: the small HDAg (S-HDAg), essential for viral replication [13], and the large HDAg (L-HDAg), crucial for virion assembly [14]. Additionally, reports suggest that L-HDAg plays pivotal roles in modulating host signaling pathways, potentially contributing to HDV pathogenesis [15]. For instance, L-HDAg has been implicated in upregulating the expression of epithelial-to-mesenchymal transition (EMT)-related proteins, which may be involved in progressive fibrosis [16].

Globally, there are eight distinct genotypes of HDV (HDV-1 to HDV-8) [17], with HDV-1 being the most widespread and prevalent. HDV-1 predominates in various regions, such as North America, Europe, and the Middle East. HDV-2 is mainly found in Asia and Russia, and HDV-4 has been exclusively identified in the Okinawa islands and Taiwan. HDV-3 is prevalent in South America, whereas HDV-5 to HDV-8 are primarily described in African populations [18]. Despite HDV genotype testing not being standard in clinical settings, emerging evidence suggests that the HDV genotype may influence disease outcomes. HDV-3 is associated with the most severe forms of hepatitis D [19], and there are reports indicating that HDV-1 is relatively more pathogenic than HDV-2 and HDV-4 in Taiwan [20]. However, the complexity of HDV genotype distribution is growing due to the increasing number of published HDV sequences and the identification of HDV RNA recombinants [21]. In regions like Asia, where multiple HDV genotypes are prevalent, HDV RNA recombination has been detected among patients with mixed-genotype infection [22]. Furthermore, numerous naturally occurring HDV recombinants have been identified through similarity analysis and recombination mapping utilizing published HDV sequences [21,23,24,25].

The fact that information regarding the direct oncogenic contributions of HDV in HCC remains limited prompted us to analyze the genetic characteristics of HDV in HCC samples obtained from the National Health Research Institutes (NHRI) Biobank in Taiwan. Our findings indicated the presence of Asian-specific HDV-2 and HDV-4 single-genotype infections, as well as HDV-2/HDV-4 mixed-genotype infections in HCC, while HDV-1 infection was not identified. Furthermore, instances of mixed-genotype HDV infections exhibited RNA recombination and novel mutations. Additionally, our investigation confirmed the direct involvement of HDV in boosting cell migration and invasion through the utilization of cloned S- and L-HDAg of HDV-2 originating from an HCC serum sample.

## 2. Materials and Methods

### 2.1. Serum Samples

The sera samples collected from 300 HBsAg-positive HCC patients were obtained from the NHRI Biobank in Taiwan on 22 November 2019. These patients tested negative for HCV and human immunodeficiency virus and had no history of intravenous drug use. Additionally, none of them exhibited autoimmune hepatitis, Wilson’s disease, hemochromatosis, or drug-induced hepatitis. Informed consent was obtained by the NHRI Biobank on 3 June 2019, and the analysis was approved by the institutional review board of the Chang Gung Medical Foundation (201801534B0 and 202101697B0). These sera were tested for anti-HDV antibody by enzyme-linked immunosorbent assay (GB HDV Ab—96T, Taiwan) according to the instructions of the manufacturers and a previous report [26]. The characteristics of patients are shown in Table S1. Of the 292 patients who tested negative for anti-HDV, the average age was 56.8 years old, male 86.3%, liver cirrhosis 38.7%, HBV DNA 389 (7–199) IU/mL, AFP level 4912 (1.52–342,193) ng/mL, and tumor size 4.926 (0.8–30) cm. The remaining 8 HCC patients were anti-HDV positive, the average age was 63.1 years old, male 87.5%, liver cirrhosis 25%, HBV DNA 196 (9–1820) IU/mL, AFP level 111.4 (3–393.5) ng/mL, and tumor size 4.725 (1.5–9) cm.

### 2.2. Analyses of HDV RNA from Serum Samples of HCC

RNA extraction from anti-HDV antibody-positive serum samples was performed using the QIAamp Viral RNA Mini Kit (QIAGEN, Hilden, Germany). Subsequently, reverse transcription was carried out with random hexamers and Moloney murine leukemia virus reverse transcriptase (Promega, Madison, WI, USA), adhering to the manufacturer’s instructions and a protocol described in a previous publication [27]. For HDV genotype determination, the synthesized cDNA underwent nested PCR using primer pairs 343 (^853^TTCGGATGCCCAGGTCGGA^871^)/344 (^1320^GGGTTCACCGACAAGGAGAG^1301^) and 18/55′ [22], amplifying a partial region of the viral genome commonly employed for HDV genotyping. The nucleotide numbering used here was based on HDV-2 TN2 clone with GenBank accession number MG557659 [27]. The PCR products were subsequently purified and directly sequenced using primer 18 [22]. Additionally, the amplified cDNA fragments were cloned into a commercial TA cloning vector (TOPO TA Cloning Vector; Invitrogen, Waltham, MA, USA). Multiple independent clones were gathered and analyzed through sequence analysis [27].

### 2.3. HDV Genome and HDAg Expression Plasmids

A full-length HDV replication clone, assembled from overlapping cDNA clones isolated from a serum sample of HDV-associated HCC 253, was constructed following procedures outlined in a previous report [27]. The complete genome sequence obtained from a serum sample of HCC 253, classified as HDV-2, was accessible in GenBank under Accession No. ON982216. The HDV-2 253 cDNA 1.2-mer was inserted into a plasmid, specifically pCR3.1, harboring a human cytomegalovirus immediate-early promoter (Invitrogen). This resultant HDV-2 253 HDV genome-expressing plasmid was denoted as pCR3.1-D_1.2_253.

The S-HDAg ORF of HCC 253 was obtained via PCR amplification using primers F4 [27] and 253N (gcggatcc-^1620^GAACTGAGGACCCTCGCCTCG^1605^), with pCR3.1-D_1.2_253 serving as the template. Subsequently, the product underwent restriction enzyme digestion and was inserted into pCR3.1, yielding the S-HDAg expression plasmid of HDV-2 253, designated as pCR3.1-Sm253. Given that the L-HDAg ORF differed from the S-HDAg ORF by only one nucleotide at the amber/W RNA editing site (A-to-G in the antigenomic RNA), the L-HDAg expression plasmid, pCR3.1-Lg253, was generated through a PCR-based site-directed mutagenesis method [28], utilizing pCR3.1-Sm253 as the template and a primer incorporating the mutation at the amber/W site.

### 2.4. Transfections and Post-Transfection Analyses

Transfection of Huh7 cells [29] was conducted using Lipofectamine 2000 (Invitrogen), following procedures described elsewhere [30]. The replication ability of the cloned HDV was assessed by transfecting HDV genome-expressing plasmids into Huh7 cells, and total cell RNA and proteins were harvested at designated time intervals post-transfection. To evaluate HDV replication in cells transfected with HDV-replicating plasmids, HDV antigenomic RNA and two forms of HDAg were analyzed via Northern blot (NB) and Western blot (WB) analyses, respectively, as described previously [27]. To investigate the impact of both HDAg forms on cell motility and host gene expression, S-HDAg- or L-HDAg-expressing plasmids were transfected into Huh7 cells, followed by selection with 400 μg/mL Geneticin (Gibco) for a duration of 2 weeks. A comparable expression level of both forms of the HDAg was confirmed by WB, and a representative result was presented in Figure S1.

### 2.5. Western Blot Assays

Total cell lysates from cultured cells were extracted by lysis buffer (150 mM NaCl/50 mMTris, pH 7.8/1% Triton X-100/1 mM EDTA, 1 mM phenylmethylsulfonylfluoride) and quantified by the Bradford method. Equal amounts of protein (20 μg) were electrophoresed through a sodium dodecyl sulfate gel of 12% polyacrylamide, and subsequently transferred to a membrane. The membranes were then probed with antibodies targeting cellular Snail (C15D3, Cell Signaling Technology, Danvers, MA, USA), Slug (C19G7, Cell Signaling Technology, Danvers, MA, USA), E-cadherin (610181; BD Biosciences, Bedford, MA, USA), N-cadherin (GTX127345, GeneTex, Irvine, CA, USA), or β-actin (T0022, Affinity bioscience, Wembley Middlesex, UK), following established protocols [31]. The antibody targeting viral proteins, including both S-HDAg and L-HDAg, was a rabbit polyclonal antibody generated against a bacterial-expressed His-tagged full-length HDAg [32].

### 2.6. Wound Healing Assay

Cell migratory capacity was assessed via a wound healing assay. Cells were cultivated in 24-well plates until they reached confluence. Then, cells were wounded using a sterilized pipet tip to make a straight scratch carefully. Afterward, any cellular debris or floating cells were cleared by washing with 1× phosphate-buffered saline (PBS), followed by the addition of a serum-free medium. The migration of cells was evaluated by comparing the percentage of wound area at specified time points to the initial wound area at 0 h. The scratched area was photographed under Cytation 1 Cell Imaging Multi-Mode Reader (Aglinet BioTek Instruments, Inc., Santa Clara, CA, USA) at selected time points and measured using the ImageJ software Image J software (version 1.52a) [33].

### 2.7. Cell Invasion Assay

The cell invasive assay was performed using 24-well Matrigel invasion chambers with 8 µm diameter pore inserts (Millipore, Temecula, CA, USA). Cells (1 × 10^5^) suspended in serum-free medium were seeded into the upper chamber, while 10% FBS was placed in the lower chamber as a chemoattractant. Following a 24 h incubation period, cells that migrated through the Matrigel-coated membrane were fixed with 4% paraformaldehyde and stained with 0.1% crystal violet solution for 10 min. Subsequently, images were acquired using an inverted microscope (IX71, Olympus, Tokyo, Japan), and quantification was conducted using ImageJ software, as previously described [33]

### 2.8. Immunofluorescence F-Actin Staining

Cells were plated onto fibronectin-coated (FC010, Millipore, Temecula, CA, USA) glass-bottom dishes (P35GC-014-C; MatTek, Ashland, MD, USA) and cultured for 24 h. After fixation with 3.7% paraformaldehyde, cells were permeabilized with 0.1% Triton X-100 in PBS for 10 min at room temperature, followed by blocking with 1% bovine serum albumin in PBS for 1 h. F-actin expression was then visualized by incubating cells with Texas Red X-Phalloidin (Invitrogen, Waltham, MA, USA), and immunofluorescence images were captured using a confocal microscope (LSM510 Meta, Zeiss, Oberkochen, Germany)). F-actin fluorescence intensity was quantified using Zen Blue edition software (version 3.2, Zeiss, Oberkochen, Germany), and intensity profiles were measured along lines spanning from the periphery to the center of the cells [33].

## 3. Results

### 3.1. RT-PCR Sequencing Analyses of HDV RNA in the Sera of HCC

**Figure 1:**
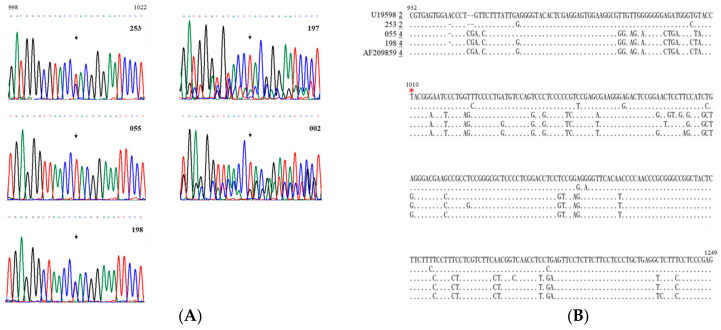
HDV-related sequences in serum samples from HCC patients. (**A**) The sequencing chromatogram spanning nucleotides 998 to 1022. Each of the DNA nucleotides adenine (A, green), cytosine (C, blue), guanine (G, black), and thymine (T, red), have their own color on the chromatogram for visualization. The sequences are summarized above the sequencing chromatograms. RNA samples obtained from patients’ sera underwent RT-PCR, followed by direct sequencing of a region encompassing the amber/W RNA editing site, marked by arrowheads. (**B**) Sequences spanning nucleotides 932 to 1249, acquired through direct sequencing of PCR products from HCC patients 253, 055, and 198, with conserved nucleotides indicated by dots. Reference isolates for HDV-2 and HDV-4 are Taiwanese T3 (GenBank accession number U19598) [34] and Taiwan-IIb-1 (GenBank accession number AF209859) clones, respectively [32]. The genotype of each clone is underlined and presented preceding the sequence. The amber/W editing site is highlighted with a red star.

Among the 300 serum samples from HBsAg-positive HCC patients tested using a commercial ELISA kit, 8 samples tested were positive for anti-HDV antibodies, indicating a positivity rate of 2.7% (Table S1). RNA extracted from these serum samples underwent genetic analysis via RT-PCR targeting a specific region spanning nucleotides 888 to 1306. Out of the eight samples analyzed, five (from patients 002, 055, 197, 198, and 253) tested positive by RT-PCR, resulting in an HDV RNA positivity rate of 62.5% among anti-HDV antibody-positive HCC patients. Subsequently, the PCR products were directly sequenced. The sequencing chromatograms covering nucleotides 998 to 1022 are illustrated in Figure 1A. Notably, two distinct sequencing patterns emerged. The first pattern, predominantly featuring single major peaks, was observed in patients 253, 055, and 198. Conversely, patients 002 and 197 exhibited a significantly more complex profile, with consistent double peaks throughout the analyzed region, suggesting a more intricate genetic composition.

### 3.2. Genotypes of HDV Isolated from the Serum Samples of HCC Patients

The genotype of HDV in individuals living with it may elevate the likelihood of liver disease progression [7,8,9,19,20]. As per the findings shown in Figure 1B, patient 253’s strain was recognized as HDV-2, whereas patients 055 and 198 were classified as HDV-4, determined by aligning HDV nucleotide sequences obtained from the direct sequencing of PCR products. Patient 253’s sequence showed a 96.6% similarity to a previously published HDV-2 sequence in a region covering nucleotides 932–1249 (GenBank accession number U19598, [34,35]. Conversely, the sequence homology between a published HDV-4 sequence (GenBank accession number AF209859, [32] and patients 055 and 198 was 96.6% and 98.1%, respectively. As illustrated in Figure 1A, the predominant peaks’ sequences identified in patients 002 and 197 within the nucleotide region 998 to 1022 also exhibited significant similarity to the sequences of HDV-4 and HDV-2, respectively. Importantly, none of these sequences belonged to HDV-1, previously associated with a less favorable disease outcome [20], including HCC, compared to HDV-2.

### 3.3. Mixed-Genotype Infections and RNA Recombination in HDV-Associated HCC

**Figure 2:**
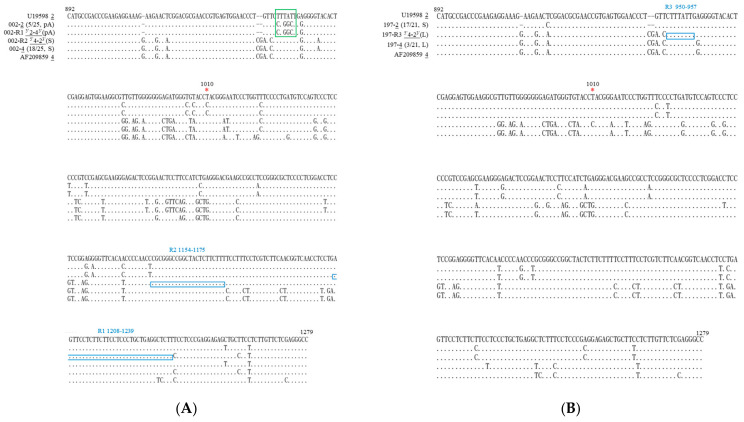
HDV-associated sequences found in patients 002 (**A**) and 197 (**B**) exhibiting mixed-genotype infection. Alignment of representative HDV sequences (nucleotides 892–1279) is presented, obtained via cloning and sequencing of PCR products. In patients 002 (**A**) and 197 (**B**), two (R1 and R2) and one (R3) recombinants were identified, respectively. The genotype of each clone is underlined. The crossover regions are indicated by blue rectangles, while the amber/W editing site is denoted by a red star. The polyadenylation signal and the sequence of the pA mutant of HDV-2 in patient 002 are depicted within a green rectangle.

To delve deeper into the molecular characteristics of HDV sequences in patients 002 and 197, PCR products were cloned into a T-vector. A sum of 25 and 21 plasmids were obtained for patients 002 and 197, respectively, and underwent sequencing analyses. As illustrated in Figure 2A, among the 25 cloned sequences from patient 002, 18 were categorized as HDV-4, while 5 were identified as HDV-2 sequences. Remarkably, we identified one ^5′^2–4^3′^ recombinant (a genomic RNA chimera comprising HDV-2 and HDV-4 sequences at its 5′ and 3′ regions, respectively) and one ^5′^4–2^3′^ recombinant (a genomic RNA chimera consisting of HDV-2 and HDV-4 sequences at its 5′ and 3′ regions, respectively). In this report, these two recombinants were denoted as R1 and R2, respectively. The junctions for the R1 and R2 recombinants were mapped to nucleotides 1208–1239 and 1154–1175, respectively. Concerning the sequences detected in patient 197 (Figure 2B), 17 cloned sequences corresponded to HDV-2, while 3 were identified as HDV-4 sequences. Furthermore, a recombinant, labeled as R3, characterized by a ^5′^4–2^3′^ configuration with a junction mapped to nucleotides 950–957, was observed. All three crossover events occurred at homologous regions between the two parental sequences (Figure 2A,B), consistent with our previous observations in a natural HDV-1/HDV-4 co-infection and in cultured cells co-transfected with HDV-1/HDV-4, HDV-1/HDV-2, and two HDV-1 sequences [22,30,36,37]. The recombination rates were 8% and 4.8% in patients 002 and 197, respectively, determined by dividing the yield of recombinants by the number of clones analyzed, akin to the 6% recombination rate observed in a previously reported natural HDV-1/HDV-4 co-infection [22]. The two recombination junctions identified in patient 002 overlapped with the crossovers identified in a patient with HDV-1/HDV-4 co-infection and in cultured cells co-transfected with HDV-1/HDV-4. The crossover identified in patient 197 (nucleotide 950–957) overlapped with a recombination junction identified in cultured cells co-transfected with two HDV-1 sequences [37,38].

In summary, out of 300 serum samples of HBV-related HCC, we observed 1 HDV-2 and 2 HDV-4 single-genotype infections, as well as 2 HDV-2/HDV-4 mixed-genotype infections. To the best of our knowledge, this represented the first report to underscore the frequent occurrence of mixed-genotype infections in HDV-related HCC. Furthermore, these data also revealed, for the first time, the occurrence of natural genetic recombination in HDV-2/HDV-4 mixed-genotype infections.

### 3.4. Mutations Leading to Codon Changes in Single-Genotype Infections of HDV

**Figure 3:**
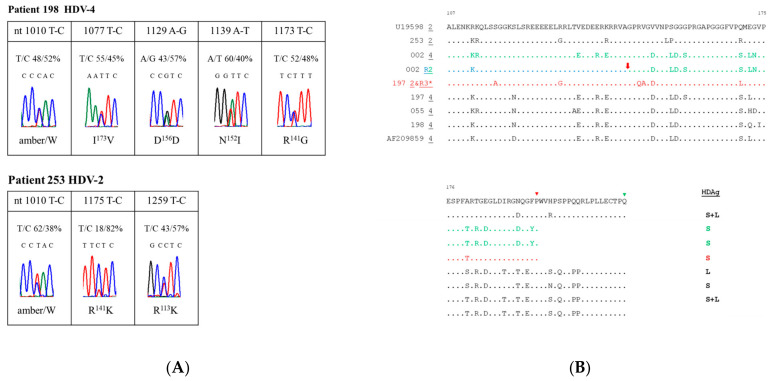
Amino acid sequences of the HDAg in the sera of HCC patients. (**A**) An overview of sequence variations, recognized as double peaks in sequencing chromatograms of patients 198 and 253. Only minor peaks, accounting for over 10% of the population, were depicted in this analysis. Percentages of specific nucleotide (nt) residues and resulting amino acid changes are summarized above and below each sequencing chromatogram. (**B**) Alignment displaying the amino acid sequences at the C-terminus of different HDAg clones discovered in five HCC patients. Each patient’s most conserved sequences are depicted, with dots indicating amino acids identical to those of the reference strain, U19598 [34]. Red arrows highlight crossover regions. The last amino acid positions for S- and L-HDAg are marked with a red and green triangle, respectively. Summaries of the HDAg variants encoded by HDV sequences associated with HCC are presented alongside the end of the amino acid sequences. *: the amino acid sequences of HDV-2 and R3 from patient 197 are identical.

Upon meticulous analysis of sequencing chromatograms derived from the direct sequencing of PCR products from individuals with single-genotype infections, it was revealed that patient 198 displayed five double peaks within the nucleotide range of 932–1249, whereas patient 253 exhibited three double peaks (Figure 3A). As anticipated, a double peak was observed at nucleotide 1010, corresponding to the amber/W RNA editing site. Sequence heterogeneity extended beyond the amber/W site, with variations detected at nucleotides 1077, 1129, 1139, and 1173 for patient 198, and at nucleotides 1175 and 1259 for patient 253. Among these sequence variations illustrated in Figure 3A, six were T-C changes, and one was an A-G change in the DNA sequencing chromatograms, likely arising from the deamination of the A residue in the antigenomic and genomic RNA, respectively. These findings also indicated that transition-type changes accumulated more frequently than transversions in the serum samples of HDV-related HCC patients. The resulting amino acid sequence variations were elaborated in Figure 3A, where, for example, variations at nucleotides 1139 and 1173 in patient 198 led to N-I and R-G changes at amino acid positions 152 and 141, respectively.

### 3.5. Novel Mutations in Mixed-Genotype Infections of HDV

Interestingly, the primary 18 HDV-4 sequences identified in patient 002 encoded S-HDAg, whereas all 5 minor HDV-2 clones underwent RNA editing at the amber/W RNA editing site (Figure 2A). Surprisingly, the minor sequences also displayed mutations at positions corresponding to the termination codon (nucleotide 952–954) for the L-HDAg. Furthermore, the UAA ochre termination codon in the L-HDAg was substituted with the CCA proline codon. Notably, the UAA termination codon was situated within the AAUAAA polyadenylation signal, known to be essential for HDV replication [39]. As shown in Figure 2A, the AAUAAA sequence was altered to AGCCAG, and the resultant mutant was labeled as the pA mutant. The ^5′^2–4^3′^ recombinant clone of patient 002 also contained the pA mutation. Consequently, it was anticipated that HDV-2 harboring such novel mutations in patient 002 would be replication-incompetent and thereby unable to produce the HDAg [39]. Hence, HDV mixed-genotype infection and subsequent RNA recombination offered a strategic mechanism for the survival of HDV carrying loss-of-function mutations.

Consistently, sequencing data from patient 197 revealed that among the 21 clones analyzed, the predominant 17 HDV-2 clones all encoded the S-HDAg, while the remaining 3 minor HDV-4 sequences expressed L-HDAg (Figure 2B and Figure 3B). However, HDV sequences carrying novel mutations destroying the polyadenylation signal were not detected in patient 197. The HDV clones with barely detectable amber/W editing rates could not efficiently undergo virion assembly by themselves, while the edited RNA genome alone failed to initiate HDV RNA replication. Therefore, mixed infection of two defective viruses was a clever strategy to maintain HDV genomes expressing S- and L-HDAg exclusively.

### 3.6. Replication of HDV-2 Clone 253 Isolated from the Serum Sample of HCC

Despite only five serum samples from 300 HCC patients ultimately testing positive for HDV RNA, unexpected yet intriguing results emerged. These findings revealed that only HDV-2 and HDV-4, previously thought to be associated with milder disease outcomes compared to HDV-1, were detected. Additionally, a significant occurrence of mixed infections of HDV-2 and HDV-4, along with homologous RNA recombination, was observed—a phenomenon that has been extensively investigated previously in our lab [21,23,30,36,37,38]. Recent meta-analysis studies have suggested HDV/HBV co-infection is a known increased risk factor for developing HCC compared to individuals with HBV mono-infection [8,9]. However, the direct involvement of HDV in HCC oncogenesis remains unclear. HDV-2 is prevalent in Taiwan and is distributed more widely geographically than HDV-4. Moreover, a full-length HDV-2-replicating clone has not yet been isolated from HCC patients. Based on these results, we decided to focus our research on the potential relationship between the HDV-2 and HCC oncogenesis. To explore this, our initial step was to obtain the full-length HDV-2 clone from the sera of HCC 253. Sequence analysis revealed that HDV-2 253 comprised 1680 bases (GenBank accession number ON982216). HDV-2 253 exhibited nucleotide sequence similarities of 95.8%, 96.5%, 80.2%, and 77.2% when compared to lab-constructed Taiwanese HDV-2 T3 (GenBank accession number U19598) [34], HDV-2 TN2 (MG557659) [27], HDV-4 Tw (AF209859) [32], and the widely used HDV-1 I (M21012; [40]), respectively. Regarding amino acid sequences, the homologies among their corresponding S-HDAg were found to be 91.8%, 93.4%, 77.4%, and 75.4%, respectively. HDV-2 253 was utilized to generate both the HDV replication clone and expression vectors for both forms of the HDAg.

**Figure 4:**
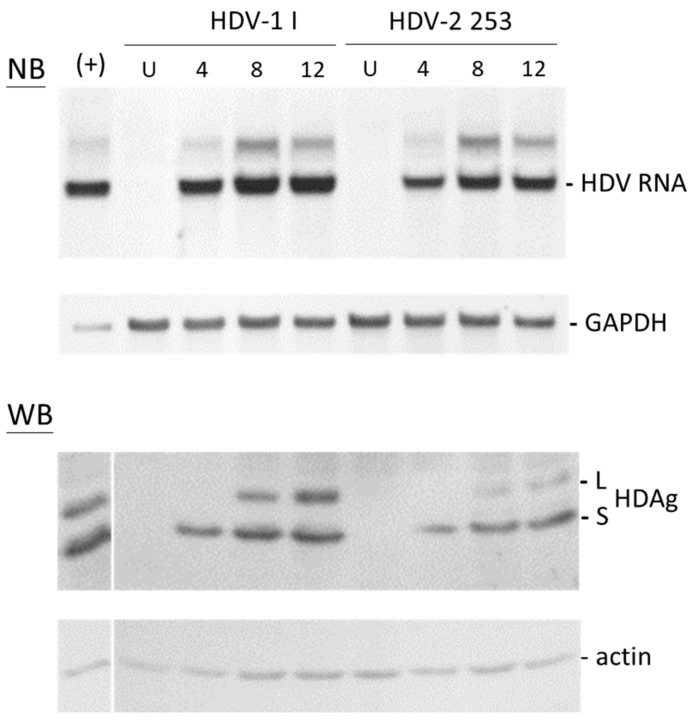
Time course of HDV-1 I and HDV-2 253 in HuH-7 cells following transfection with a genome expression plasmid. RNA and proteins were collected at specified intervals post-transfection. Analysis of HDV antigenomic RNA and HDAg was conducted by NB and WB assays, respectively. The uniform loading of RNA and protein samples was verified through hybridization with a glyceraldehyde-3-phosphate dehydrogenase (GAPDH) probe and an actin-specific antibody, respectively. U: untransfected cells.

Notably, our previous studies found HDV-2 T3 and TN2 RNA to be scarcely detectable in transfected Huh7 cells [27,35]. To evaluate the replicative capacity of HDV-2 253, HDV-replicating plasmids were introduced into HuH7 cells. The findings presented in Figure 4 demonstrated that the antigenomic RNA levels of both HDV-1 I and HDV-2 253 peaked on day 8 and remained stable until day 12. Additionally, both forms of HDAg from HDV-1 I and HDV-2 253 were detectable at days 8 and 12, with L-HDAg accumulating throughout the observed time points. Quantitative analysis of HDV RNA revealed that HDV-2 253 could replicate up to 58%, 67%, and 74% of the level observed for HDV-1 I at 4, 8, and 12 days post-transfection, respectively, significantly exceeding the replication capability of previously published HDV-2 clones T3 and TN2. Furthermore, comparing the replication levels between day 4 and day 12 post-transfection, an increase of 13% and 45% in HDV RNA levels was observed for HDV-1 I and HDV-2 253, respectively. Thus, although HDV-2 153 initially replicated less efficiently than HDV-1 I at day 4 post-transfection, it accumulated at a faster rate at later time points. Concerning the production of the HDAg, L-HDAg accounted for 38% and 23% of the total HDAg at 12 days post-transfection for HDV-1 I and HDV-2 253, respectively.

### 3.7. Both Forms of the HDAg Facilitate Cell Migration and Invasion

**Figure 5:**
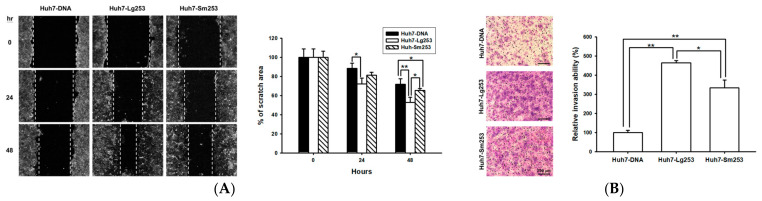
Impact of HCC serum-derived Sm253 and Lg253 expression on cell migration and invasion in Huh7 cells. (**A**) Both Sm253 and Lg253 promote cell migration. Huh7 cells overexpressing either Sm253 or Lg253 were evaluated through a wound healing assay. Control cells were transfected with a vector. Images were captured at 0, 24, and 48 h, with the dotted line denoting the average leading edge of cellular migration. ImageJ software was utilized to measure the wound area, and the data are presented as mean percentages (±SE; *n* = 4) relative to the control cells. (**B**) Transwell invasion assays were conducted to assess the invasion capacities of Sm253 and Lg253. Images were acquired at 24 h and a representative image from three independent experiments was displayed. Data are depicted as mean percentages (±SE, *n* = 3) compared to the control cells. * indicates *p* < 0.05, ** indicates *p* < 0.01.

Prior studies have indicated that L-HDAg, in contrast to S-HDAg, plays a pivotal role in modulating host signaling pathways and regulating EMT, potentially contributing to HDV pathogenesis [15,16]. To further explore this phenomenon, plasmids expressing two forms of the HDAg derived from HDV-2 253, denoted as Sm253 and Lg253, were generated and then subjected to wound healing migration assays (Figure 5A). Notably, after 48 h of scratching, the progression of wound healing was significantly accelerated in Huh7 cells expressing Lg253 and Sm253 in contrast to control Huh7 cells, suggesting an enhanced migration ability in both forms of the HDAg (Figure 5A). Furthermore, the findings from the wound healing migration assays revealed that Huh7 cells expressing Lg253 displayed an even higher migration capability than those expressing Sm253. Similar trends observed in cell invasion assays indicated that not only did Lg253 (showing a 4.6-fold increase)- but also Sm253 (showing a 3.4-fold increase)-expressing Huh7 cells displayed a significantly enhanced invasion ability across Matrigel compared to the control Huh7 cells (Figure 5B). These findings represent the first evidence that the expression of both forms of the HDAg substantially enhances the cell migration and invasion capability.

### 3.8. HDAg Boosts Cell Motility and Regulates the Expression of EMT Markers

**Figure 6:**
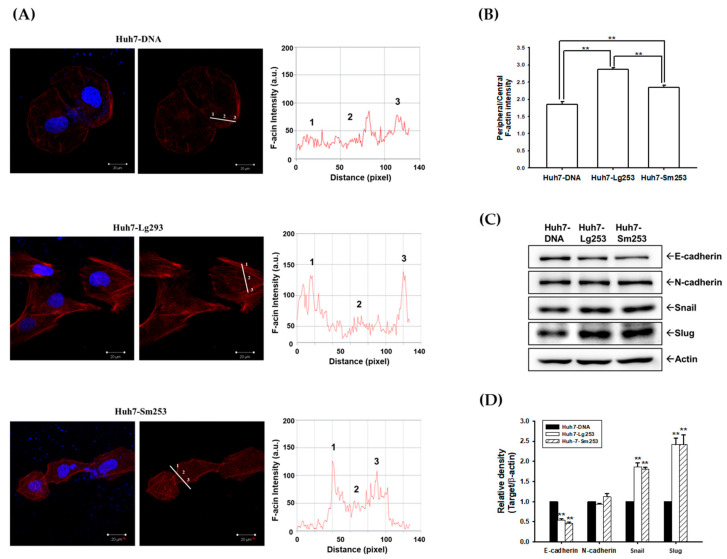
Sm253 and Lg253 modulate the F-actin distribution and expression of EMT markers. (**A**) The F-actin was stained using Texas Red X-Phalloidin, with the nuclei counterstained with DAPI (blue), and the immunofluorescence was captured using a confocal microscope. (**B**) The quantitative analysis of the F-actin fluorescence intensity. The intensities were measured along the line from the peripheral to the center of the cells, and the quantitative analyses of the F-actin fluorescence intensity of control cells and cells expressing Lg253 and Sm253 are shown (± SE, n = 6). ** indicates *p* < 0.01. (**C**) Immunoblot assays determined E-cadherin, N-cadherin, Snail, and Slug protein levels. (**D**) The quantitative analyses of the immunoblots are presented as a relative density of EMT markers. Actin protein level was determined to compare the total protein levels in each lane. ** indicates *p* < 0.01.

We proceeded to assess cell motility by examining actin cytoskeleton rearrangements. Using Texas Red X-Phalloidin to stain cellular F-actin, fluorescence intensity analysis demonstrated that both Lg253- and Sm253-expressing Huh7 cells exhibited increased peripheral F-actin distribution compared to the control Huh7 cells, suggesting that overexpression of Lg253 or Sm253 prompted actin polymerization and enhanced cell motility (Figure 6A). Additionally, Lg253-expressing Huh7 cells displayed higher levels of F-actin polymerization compared to cells expressing Sm253 (Figure 6B). Consistently, immunoblot assays confirmed that both Lg253 and Sm253 overexpression resulted in increased levels of Snail and Slug proteins, while simultaneously reducing E-cadherin expression in Huh-7 cells. However, the expression of N-cadherin remained unaffected by both Sm253 and Lg253 (Figure 6C,D).

## 4. Discussion

HDV, a subviral satellite of HBV, adds complexity to delineating the distinct roles each virus plays in hepatocarcinogenesis. Moreover, uncertainties persist regarding the attributes of HDV within HBV/HDV-related HCC, primarily due to challenges in obtaining HCC samples exhibiting serological signs of HDV infection. This report concentrates on examining the molecular characteristics of HDV in the sera of HCC, aiming to illuminate its oncogenic involvement in HCC tumorigenesis.

This report estimates the prevalence of anti-HDV antibodies among individuals who test positive for HBsAg to be 2.7%, in accordance with the results of a recent publication [6]. It is widely acknowledged that in Taiwan, HDV-2 (38.1%, 74/194) is the predominant genotype, followed by HDV-1 (26.3%, 51/194), with HDV-4 constituting only 4.1% (8/194) (61 represents an unclassified HDV genotype) [20]. Furthermore, the severity of the disease exhibits a stronger correlation with HDV-1 in contrast to HDV-2 and HDV-4 [20,41]. Recently, the spread of viral hepatitis among injecting drug users (IDUs) is becoming an increasingly significant public health issue. Reports have shown that HDV-2 and HDV-4 are becoming the predominant genotypes, with HDV-1 representing only a small portion of HDV infections among IDUs in Taiwan [42,43]. Our findings revealed that HDV-2 and HDV-4, previously linked with milder illnesses, were present in all 5 HCC cases identified among 300 serum samples associated with HBV-related HCC. Therefore, the accuracy of the genotype distribution and the correlation between HDV genotype and HCC formation require larger sample sizes and rigorous analyses to draw precise conclusions. In the future, the emergence of HCC in populations infected with HDV-2 and HDV-4, previously believed to be linked with milder diseases, may warrant increased attention and monitoring.

The inability to detect RNA editing in sample 005 is a perplexing but not uncommon occurrence. According to a previous report, even when using highly sensitive next-generation sequencing methods to study HDV RNA editing in serum samples from HDV patients, it was found that among 219 serum samples, the editing rate of 3 samples repeatedly fell between the lower limit of detection (1%) and the lower limit of quantification (5%) [44]. The NHRI biobank provides only a single time point for sample collection. However, based on our unpublished data analyzing the RNA editing rate from multiple time points using serum samples from chronic hepatitis D patients, if RNA editing is undetectable at one time point, measuring RNA editing at preceding and subsequent time points reveals that RNA editing indeed occurs. Therefore, examining the fluctuations in the HDV RNA editing rate within the serum samples and infected liver tissues of HCC should provide a clearer understanding of the variability in HDV sequences, including RNA editing rates.

To our knowledge, mixed-genotype infections of HDV have only been documented in patients in Taiwan. Previously, mixed-genotype infections, such as HDV-1/HDV-2, HDV-2/HDV-4, and HDV-1/HDV-4, have been documented [22,42,45,46]. These data suggested that mixed-genotype infections occurred frequently among high-risk groups, such as prostitutes and their sexual contacts as well as human immunodeficiency virus positive IDUs [42,45]. However, such mixed-genotype infections were not observed in many HBV/HDV co-infected Taiwanese patients with varying disease outcomes [20,47]. Among them, HDV recombination was only detected in a single instance of HDV-1/HDV-4 mixed-genotype infection [22]. Nonetheless, numerous HDV RNA recombinants have been identified through similarity analysis and recombination mapping using published HDV sequences [21,23,24,25]. Since HDV RNA recombination is homologous, it is reasonable to expect that mixed-strain infections of the same HDV genotype are not uncommon occurrences, necessitating careful examination of HDV-related sequences in patients, which could also complicate epidemiological data. In this study, RNA recombination was also identified in two cases of HDV-2/HDV-4 mixed-genotype infections among five HDV RNA-positive HCC patients. The frequent occurrence of mixed-genotype infections in HDV-related HCC patients suggests the possibility that such infections, along with the sequence diversity they introduce, could contribute to more complex virus–host interactions, potentially promoting carcinogenesis. However, this hypothesis needs further validation through additional research.

The mutations discovered in sample 002 not only changed the termination codon for the L-HDAg but also disrupted the polyadenylation signal for the HDAg. Previous studies have shown that when the polyadenylation signal is disrupted, HDV loses its ability to replicate [39]. The identification of a replication-deficient viral sequence in the serum of HCC patients raises the intriguing question: why would a virus generate mutations that are detrimental to its own replication function, and yet these mutations are retained? It is indeed an intriguing phenomenon. The most plausible explanation for the survival of this variant viral sequence is RNA recombination. Whether this mutant strain possesses other functions that could promote hepatocarcinogenesis after recombination is a crucial topic for future investigation. There is a possibility that despite the mutation of the polyadenylation signal, the virus can still generate polyadenylated mRNA. This notion is supported by previous research suggesting that polyadenylation may still occur at downstream self-cleavage sites despite mutations at the polyadenylation site [48].

More and more countries have demonstrated the presence of multiple genotypes of HDV in circulation. For example, HDV-2b and HDV-8 have been detected in Egypt and in Brazil, respectively [49,50]. A recent report indicates the presence of HDV-1, 2, and 5 in Kyrgyzstan [21], a country in Central Asia. Additionally, immigrants originating from areas where HDV is endemic significantly add to the HDV infection burden and introduce various African genotypes into Europe [51,52,53]. Consequently, the impact of HDV RNA recombination on the epidemiology, evolution, prevention, and clinical management of HDV infection warrants increased attention.

Interestingly, in the two mixed-genotype infections presented in this report, the major sequence identified encoded S-HDAg, while the minor sequence translated L-HDAg (Figure 2A and Figure 3B). While these two kinds of HDV individually lacked the ability to survive in nature, their coexistence likely facilitated successful RNA replication and HDV virion production. Furthermore, subsequent RNA recombination events likely added to the genetic complexity of HDV in HCC. Given that only 25 or 21 cloned sequences for the serum samples of HCC 002 and HCC 197, respectively, were analyzed here, the possibility of the existence of functional forms of the HDAg for each virus in the mixed-genotype infections cannot be discounted. Furthermore, conducting analysis on a greater number of HDV sequences from mixed-genotype infections would provide further understanding of the resulting HDV chimeras and their potential biological functions and pathogenicity. However, a significant limitation of our study is the lack of sequence data covering the entire HDAg ORF, especially those containing novel mutations corresponding to the polyadenylated signal of the HDAg. The size, sequences, and biological functions of the resulting HDAg mutants and chimeras raise intriguing questions that require further exploration.

The majority of published HDV-replicating clones are derived from chronic hepatitis patients or experimental animals. To our knowledge, only one HDV-4-replicating clone has been obtained from an HCC patient [54]. In this study, we successfully constructed the first HDV-2-replicating clone derived from the serum sample of an HCC patient. Regarding the replication capacity of various published HDV-2 clones, we focused solely on reports providing comparative data to the widely used HDV-1 I clone, enabling us to assess the relative replication levels of HDV-2 across different laboratories. Previously isolated HDV-2 clones, T3 and TN2, exhibited much less efficient replication in cultured cells compared to HDV-1 I [27,35]. Recently, replicating clones of HDV of all genotypes have been documented, with HDV-2, identified by the GenBank accession number X60193, demonstrating the lowest level of replicative RNA among the eight genotypes analyzed [55]. In contrast to prior findings, HDV-2 253, isolated from an HCC patient, exhibited a replication ability up to 74% of the level observed for HDV-1 I at 12 days post-transfection. The potential contribution of this relatively higher replication level of the HDV-2 253 clone to the observed pathogenesis remains uncertain.

It has long been recognized that L-HDAg, rather than S-HDAg, regulates host signaling pathways, thereby contributing to HDV pathogenesis. Reports have suggested that L-HDAg activates the serum response factor and serum response element pathways, which then transactivates the c-fos promoter [56]. Moreover, L-HDAg is involved in the up-regulation of TGF-β- and c-Jun-dependent signaling cascades as well as in the TNF-α-induced NF-κB transcriptional activation in a cultured cell model [57,58]. There is also a report that indicates that L-HDAg increases the production of reactive oxygen species via NADPH oxidase-4 and affects STAT3 and NF-kB signaling [59]. Moreover, L-HDAg increases the expression of the epithelial-to-mesenchymal transition (EMT)-related proteins via TGF-β/Smad3/Twist activation, which might be involved in progressive fibrosis [16,54]. Despite the accumulated data from these studies, many details are still missing on the network of signaling cascades triggered by HDV. One major reason could be that the HDAg used in these studies was not isolated from patients with HCC. Our findings revealed that the overexpression of both Sm253 and Lg253 isolated from an HCC patient in Huh-7 cells facilitated cell migration and invasion, with Lg253 demonstrating a stronger inducer for these processes. Furthermore, both forms of HDAg induced F-actin remodeling and influenced the expression of EMT markers, indicating a pivotal role in cellular motility and invasion. The C-terminal prenylation domain of L-HDAg plays a crucial role in activating certain host signaling pathways [16,57,59]. The absence of a prenylation signal on Sm253 implies that domains within the first 195 amino acids also participate in regulating host gene expression. Indeed, it has been shown that the N-terminal region of HDAg could interact with Smad3 [57]. The exact mechanisms shared or uniquely utilized by two forms of the HDAg in the context of HCC oncogenesis are intriguing and warrant further investigation.

## Data Availability

The genome sequence of HDV-2 253 presented in this study is openly available in GenBank under accession number ON982216.

## Supplementary Materials

The following supporting information can be downloaded at: https://www.mdpi.com/article/10.3390/v16060817/s1, Table S1: comparisons of patient characteristics, diagnostic features, treatment, and outcomes of HBV-associated HCC diseases. Figure S1: illustration of the efficiencies of HDAg overexpression in Huh7 cells, as determined by analyzing HDAg protein expression via WB.
