# Supplementary material for: Investigating the Genetic Diversity of Hepatitis Delta Virus in Hepatocellular Carcinoma (HCC): Impact on Viral Evolution and Oncogenesis in HCC
Source: Viruses. 2024 May 21;16(6):817. doi: 10.3390/v16060817 (PMC11209585; doi:10.3390/v16060817)
Supplement: Supplementary file 1 [file viruses-16-00817-s001.zip › viruses-2950873-supplementary.pdf]

Table S1: Comparisons of patient characteristics, diagnostic features, treatment, and outcomes of HBV-associated HCC diseases

	All patients(n=300)	Anti-HDV Negative (n=292)	Anti-HDV positive (n=8)
Male, *n*(%)	259(86.3)	252(86.3)	7(87.5)
Age, years	57(18-90)	56.8(18-90)	63.1(55-79)
AFP	4,780(1.52-342,193)	4,912(1.52-342,193)	111.4(3-393.5)
tumor size	4.921(0.8-30)	4.926(0.8-30)	4.725(1.5-9)
Albumin	4.226(2.6-5.3)	4.23(2.6-5.3)	3.36(3.4-4.56)
ALT(U/L)	35.8(9-154)	35.993(9-30)	30.875(9-49)
Bilirubin(mg/dl)	0.773(0.2-3)	0.781(0.2-0.7)	0.475(0.2-1)
HBV DNA($\times 10^6$ IU /ml)	0.000389(0.000007-0.00199)	0.000389(0.000007-0.00199)	0.000196(0.000009-0.00182)
HBeAg(+),*n*(%)	9(3)	9(3)	0
Smoking history(+), *n*(%)	158(52.67)	152(52.05)	6(75)
Drinking history(+), *n*(%)	123(41)	117(40.07)	6(75)
Cirrhosis(+), *n*(%)	115(38.33)	113(38.7)	2(25)
Metastasis(+), *n*(%)	15(5)	15(5)	0
HCC type [a], *n*(%)			
1	231(77)	224(76.7)	7(87.5)
2	69(23)	68(23.3)	0
Vascular invasion [b], *n*(%)			
0	146(48.7)	143(49)	3(37.5)
1	51(17)	48(16.4)	0
2	65(21.7)	65(22.3)	4(50)
3	4(1.3)	0	0
4	21(7)	21(7.2)	0
1,2	14(4.7)	14(4.8)	0
1,3	1(0.3)	1(0.3)	1(12.5)

1,4	1(0.3)	1(0.3)	0
Pathology stage, *n*(%)			
I	112(37.3)	110(37.7)	2(25)
II	117(39)	113(38.7)	4(50)
IIIA	22(7.3)	21(7.2)	1(12.5)
IIIB	32(10.7)	31(10.6)	1(12.5)
IIIC	7(2.3)	7(2.3)	0
IVA	3(1)	3(1)	0
IVB	7(2.3)	7(2.3)	0
Treatment before surgery [c], *n*(%)			
0	260(86.7)	252(86.3)	0
1	16(5.3)	16(5.5)	0
2	12(4)	12(4.1)	0
3	6(2)	6(2.1)	0
4	2(0.7)	2(0.7)	0
5	2(0.7)	2(0.7)	0
6	1(0.3)	1(0.3)	0
7	1(0.3)	1(0.3)	0
HDV Genotype			
HDV-2, *n*	1	0	1
HDV-4, *n*	2	0	2
HDV-2/HDV-4, *n*	2	0	2
ND [d], *n*	3	0	3

a. HCC type: 1. Solitary; 2. Multiple

b. Vascular invasion: 0. Absent ; 1. capsular vein invasion; 2. portal vein tumor thrombosis (micro); 3. portal vein tumor thrombosis (grossly); 4.portal vein tumor thrombosis (gross and micro)

c. Treatment before surgery"no"=0 "TAE/TACE"=1 "Surgical"=2 "RFA"=3 "TAE/TACE,RFA"=4 "chemotherapy"=5 "TAE/TACE,RFA,cryotherapy"=6 "TAE/TACE,PEI/Alcohol injection”7”=missing data

d. ND: HDV RNA undetectable

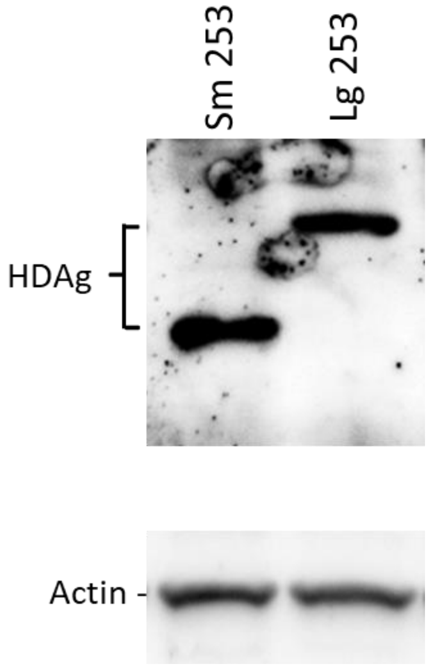

Figure S1. Illustration of the efficiencies of HDAg overexpression in Huh7 cells, as determined by analyzing HDAg protein expression via WB. Sm 253 and Lg 253 denote S-HDAg and L-HDAg, respectively, derived from the serum sample of HCC 253.
